# Role of Preschool and Primary School Children in Epidemics of Influenza A in a Local Community in Japan during Two Consecutive Seasons with A(H3N2) as a Predominant Subtype

**DOI:** 10.1371/journal.pone.0125642

**Published:** 2015-05-05

**Authors:** Satoshi Mimura, Taro Kamigaki, Yoshihiro Takahashi, Takamichi Umenai, Mataka Kudou, Hitoshi Oshitani

**Affiliations:** 1 Department of Virology, Tohoku University Graduate School of Medicine, Sendai, Japan; 2 Division of Pediatrics, Odate Municipal General Hospital, Odate, Japan; 3 Umenai Pediatric Clinic, Odate, Japan; 4 Kudou Pediatric Clinic, Odate, Japan; Harvard School of Public Health, UNITED STATES

## Abstract

Enhanced influenza surveillance was implemented to analyze transmission dynamics particularly driving force of influenza transmission in a community during 2011/12 and 2012/13 seasons in Odate City, Japan. In these two consecutive seasons, influenza A(H3N2) was the predominant influenza A subtype. Suspected influenza cases were tested by commercial rapid test kits. Demographic and epidemiological information of influenza positive cases were recorded using a standardized questionnaire, which included age or age group, date of visit, date of fever onset, and the result of rapid test kit. Epidemiological parameters including epidemic midpoint (EM) and growth rate (GR) were analyzed. In 2012/13 season, numbers of influenza A positive cases were significantly lower among preschool (212 cases) and primary school (224 cases) children than in 2011/12 season (461 and 538 cases, respectively). Simultaneously, total influenza A cases were also reduced from 2,092 in 2011/12 season to 1,846 in 2012/13 season. The EMs in preschool and primary school children were earlier than EMs for adult and all age group in both 2011/12 and 2012/13 seasons. The GR in 2012/13 season was significantly lower than that in 2011/12 season (0.11 and 0.18, respectively, p = 0.003). Multiple linear regression analysis by school districts revealed that GRs in both seasons were significantly correlated with the incidence of school age children. Our findings suggest that preschool and primary school children played an important role as a driving force of epidemics in the community in both 2011/12 and 2012/13 seasons. The reduction of total influenza A cases in 2012/13 season can be explained by decreased susceptible population in these age groups due to immunity acquired by infections in 2011/12 season. Further investigations are needed to investigate the effect of pre-existing immunity on influenza transmission in the community.

## Introduction

Influenza is an important public health issue not only because a novel influenza virus can cause a pandemic but also there is a significant impact due to seasonal influenza outbreaks. It is estimated that 250,000 deaths due to seasonal influenza occur every year in the world [1]. It is necessary to understand the epidemiology of influenza including transmission dynamics to implement more effective prevention and control strategies such as vaccination and social distancing. Transmission dynamics of influenza in a community can be affected by many factors including demographic characteristics, immune status of the population, and social contact patterns [2].

Currently two influenza A subtypes, namely A(H3N2) and A(H1N1)pdm09 are circulating in human populations. We experienced two consecutive influenza seasons with influenza A(H3N2) as the predominant influenza A subtype in the 2011/12 to 2012/13 seasons. Predominant circulation of influenza A(H3N2) for these two seasons were also documented in other countries such as France [3]. A study from the Netherlands revealed a significant contribution of depletion of susceptible population to the trend of influenza-like illness epidemics across seasons [4]. A surveillance study in Italy found a decline of transmissibility in children but an increase in adults during consecutive seasons with influenza A(H3N2) epidemics [5]. A cohort study in Hong Kong revealed that prior infections with influenza A(H3N2) reduced the risk of repeated influenza A(H3N2) infections in subsequent epidemics among school-age children [6].

School-age children often play an important role as a driving force for transmission of seasonal influenza [7,8] and pandemic influenza [9] in the community. This may be because children may not have pre-existing herd immunity [9] or because they have higher intensity of social contacts [10]. The national influenza surveillance in Japan reports annual total influenza cases by age groups. According to these reports, school-age children (5–9 years and 10–14 years) comprise around 40% of total influenza cases in normal seasons. However, the proportion of cases in these age groups was significantly lower (27%) in 2012/13 season [11]. Therefore, we compared the epidemiological characteristics of influenza A between 2011/12 and 2012/13 seasons by analysing the data obtained from an enhanced influenza surveillance in Odate City, Japan to define transmission dynamics including driving forces of influenza A transmission in a community within the two consecutive seasons.

## Materials and Methods

### Study site and influenza surveillance

The study was conducted in Odate City, which is located in Akita Prefecture in the northeast of Honshu, the main island of Japan. Odate City is a middle-sized city in a rural area of Japan with a total population of 78,946 according to the 2010 national census. We used this population data in the analysis. The proportions of people aged < 20 and > 65 years old are 15.6% and 31.7%, respectively. In Odate City, more than 35 clinics and 7 hospitals provide outpatient care. An enhanced influenza surveillance has been carried out since November 2011 in 22 clinics, one emergency clinic and one outpatient clinic in a general hospital [12]. These 24 outpatient clinics, which can provide diagnosis and treatment of influenza cases, participated in the study voluntarily. Every patient with a suspected influenza infection was tested using commercially available rapid diagnostic kits, which is a common practice in Japan. Different rapid diagnostic kits were used in different clinics but all diagnostic kits used could differentiate influenza A and B infections. A standardized questionnaire was distributed to the participating clinics for collecting demographic and epidemiological information from influenza diagnostic kit-positive cases. Collected information included patient initials, gender, age or age group, date of visit, date of fever onset, name of clinic, the result of rapid test kit, affiliation (school and class) or occupation, address, presumptive source of infection, and influenza vaccination status. Influenza vaccination status was collected only for 2012/13 season. We excluded influenza positive cases whose residential addresses are outside of Odate City from the analysis.

All data were stored in a database and only influenza A positive cases identified from November 1, 2011 to October 31, 2013 were analyzed. The 2011/12 season spans the period from November 1, 2011 to October 31, 2012 and the 2012/13 season from November 1, 2012 to October 31, 2013.

The national surveillance of infectious diseases reported nine strains of influenza A(H1N1)pdm09 and 3,706 strains of influenza A(H3N2) in 2011/12 season and 152 influenza A(H1N1)pdm09 and 4,973 influenza A(H3N2) in 2012/13 season, indicating that the majority of influenza A viruses were influenza A(H3N2). Antigenic analysis of influenza A(H3N2) strains in Japan showed that most strains in 2011/12 season were antigenically similar to the vaccine strain, A/Victoria/361/2011 [13], and 99% of the strains in 2012/13 season were also similar to A/Victoria/361/2011 [14].

### Defining age group

Patients’ age were distributed into five groups: preschool, primary school, junior high school, high school, and adults. Preschool group was defined as those aged less than school-age. High school group included those who were aged between 16 and 18 years old regardless of their actual status of attending school. In the regression analysis, three groups including junior high school, high school and adults were merged.

### Epidemic midpoint and growth rate

The epidemic midpoint (EM) was defined as the date when cumulative incidence reached 50% of the total cumulative cases in each season [15]. The EM is considered to indicate the timing of the peak of influenza activity. To compare the relative timing of peak influenza A activity in each age group, the lead time was calculated as the difference between the EM of each age group and the overall EM for seasons. We set EM in 2011–2012 season as day 0 to visualize the timing of EM of each age group.

In an early phase of an infectious disease epidemic including influenza, increase of cases is commonly assumed to follow an exponential growth [16]. The growth rate (GR) is a parameter to express this exponential growth and widely used to estimate a reproduction number, which is one of the key parameters to express influenza transmissibility [17–19]. In this analysis, the exponential period for each season was defined between the onset day of the first case detected in surveillance and the EM of all age. The GR was calculated during this period. The formula for growth rate (r) of the cumulative number of cases is

$$C\left(t\right)=ke^{r\left(t-\tau \right)}$$

where C(t) is the cumulative number of cases on day t, r is the growth rate, and τ is date when an exponential growth is assumed to have started. We defined that τ for each season was the day when the first influenza A case among all age groups reported fever onset.

### Statistical analysis

The area of the city is divided into 18 primary school districts and children who live in sub-districts within the school zone usually go to the designated primary school. We obtained the map of the school districts from the national spatial planning database [20]. We corresponded the number of population at sub-district level in national census database to the school district level by matching the address of sub-districts to school districts. In case that more than one school district is in one sub-district, the number of population was determined by the proportion of the school districts’ areas.

We assumed that the population at risk in the 2011/12 season was the whole population in each age group and that those at risk in the 2012/13 season was calculated by subtracting the total number of influenza A cases in the 2011/12 season from the whole population in each age group.

We then conducted linear regression analysis to investigate the association of the GRs of each primary school district with the incidences of influenza infection of different age groups and with the depletion of susceptible population in primary school for the 2012/13 season. We simply assumed the number of the primary school children cases that were positive for influenza A in 2011/12 season illustrated a majority of the depletion of susceptibles in that age group. As described before, GR is a parameter of transmissibility in exponential phase of epidemics and previous study [8] as well as our study showed relative accumulation of both primary school and preschool children in that phase so that we set this regression analysis. With our linear regression model, where GRs calculated in each school district were a dependent variable and the incidences of age groups were independent variables, we first applied the model with incidences of preschool, primary school, and other age groups as independent variables for each season (model 1 for 2011/12 season and 2012/13 season). Additionally, we included the total number of influenza A cases in 2011/2012 season as one of the independent variables for GR in the 2012/2013 model (model 2 for 2012/13 season). Model 1 was a single year reproduction model. Model 2 was a consecutive year reproduction model especially for primary school children. All 18 primary school districts in Odate City were included in both models. The variance inflation factor (VIF) of variables in these models were calculated for the quantification of multicollinearity among those independent variables. A VIF of more than 10 was set as a significant multicollinearity.

Chi-square test was used to compare proportion of influenza A positive cases to the total population. The analysis was done with JMP pro 11 (SAS Institute Japan) and R 3.1.0 (R Development Core Team).

### Ethical consideration

We distributed the statement of the study that include the details of our contact to all study participants. For those aged less than 18 years, the written informed consent was obtained from their guardians. For participants of over 18 years, the consent was initially obtained verbally. After obtaining consent, the standardized questionnaire was filled. In case of the refusal to participate, that questionnaire was discarded and never included in the database. This study was approved by the Ethics Committee of Tohoku University Graduate School of Medicine (ID 2011–268).

## Results

**Fig 1:**
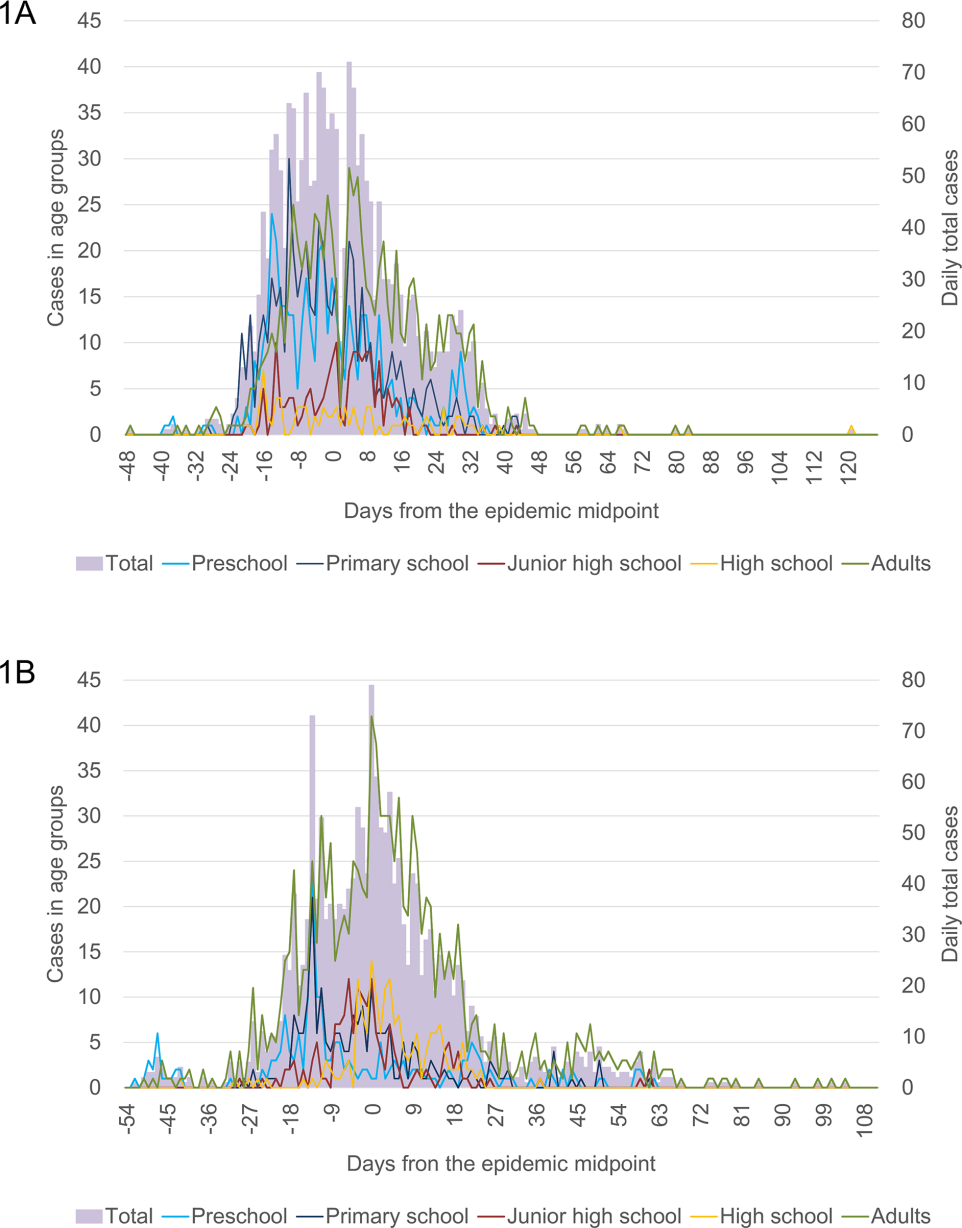
Daily number of Influenza A positive cases by age group in 2011/12 (1A) and 2012/13 (1B) seasons in Odate City, Akita, Japan. * The day of epidemic midpoint was set as Day 0. Those dates were February 8, 2012 and February 2, 2013, respectively.

There were a total of 2,092 and 1,846 influenza A cases in 2011/12 and 2012/13 seasons, respectively. The proportion of influenza A positive cases in the total population in the 2011/12 season was significantly higher than that in the 2012/13 season (p<0.001). The daily number of influenza A cases by age group was plotted against the days from the epidemic midpoint for the two seasons (Fig 1). Overall temporal trends of influenza A positive cases in the 2011/12 and the 2012/13 seasons were similar. However those in different age groups showed apparent differences between seasons. In the 2011/12 season, numbers of cases in preschool and primary school were high almost throughout the epidemic period. But in the 2012/13 season, higher numbers of cases in these age groups were seen only until day -10 from EM and they occupied small portion of total cases during the peak period. Number of cases in high school was quite low throughout the 2011/12 season but they formed a clear peak in the 2012/13 season on the day of EM. Temporal trends of adult cases in the two seasons were similar.

The percentage of positive influenza cases in preschool was 22.0% and the percentage in primary school was 25.7% in the 2011/12 season (Table 1).

**Table 1:** Demographic and epidemiological characteristics of influenza A positive cases in 2011/12 and 2012/13 seasons.

Categories	Population	2011/12 season		2012/13 season	
		No. (%)	AR (%)	No. (%)	AR (%)
Total	78,980	2,092 (100)		1,846(100)	
Age group*					
Preschool	3,636	461 (22.0)^1^	12.6	212(11.5) ^1^	5.8
Primary School	3,664	538 (25.7)^2^	14.7	224(12.1) ^2^	6.1
Junior High School	2,032	169 (8.1)^3^	8.3	160(8.7) ^3^	7.9
High School	2,179	91 (4.3) ^2^	4.2	178(9.6) ^2^	8.2
Adults	67,469	833 (39.8) ^2^	1.2	1072(58.1) ^2^	1.5
Sex					
Female	42,112	1010 (48.3)	2.4	913 (49.5)	2.2
Male	36,868	1082 (51.7)	2.9	933 (50.5)	2.5

* The size of population in each age group was estimated with data of September, 2012

The p-value was calculated based on the chi-square distribution.

1. p = 0.002

2. p < 0.001

3. p = 0.507

However, their respective proportions declined significantly to 11.5% (p = 0.002) and 12.1% (p<0.001) in the 2012/13 season. On the other hand, the total numbers of positive cases in adults and high school were 1.29 and 1.96-fold higher in the 2012/13 season than in the 2011/12 season (p < 0.001, both). The numbers of cases in junior high school was similar between the two seasons.

**Fig 2:**
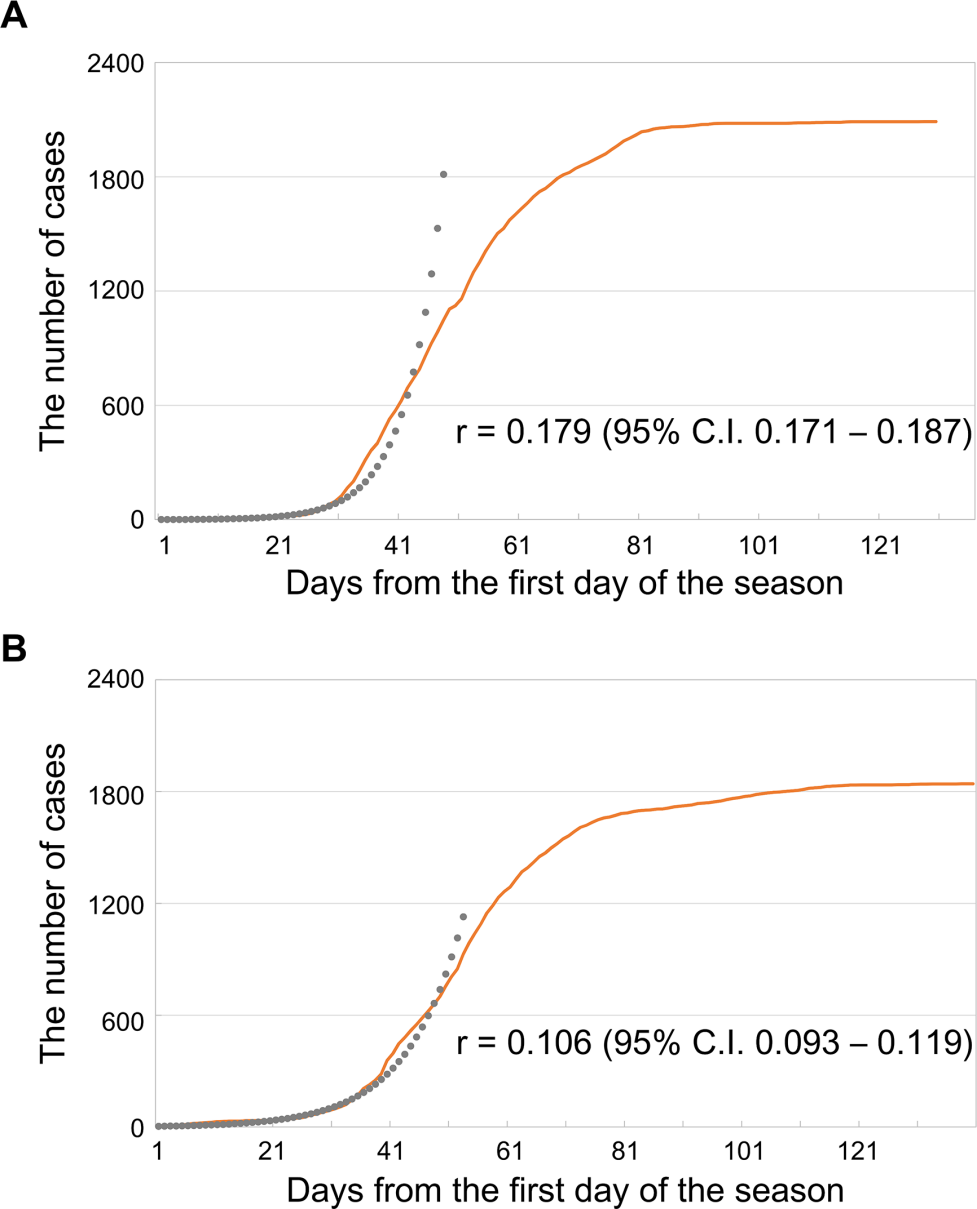
Cumulative number of influenza A cases and growth rate (r) in 2011/12 (A) and 2012/13 (B) seasons. * The first days of the season were December 23 in 2011 and December 12 in 2012.

The growth rate (GR) of 0.179 in the 2011/12 season was significantly higher than the GR of 0.106 in the 2011/12 season (p = 0.003) (Fig 2). The exponential period for the 2011/12 season and the 2012/13 season were 48 and 53 days, respectively. There was more influenza A positive cases within the exponential period in preschool and primary school children in the 2011/12 season than in the 2012/13 season (Table 2) while in junior high school and high school students, and adults there were more cases in the 2012/13 season than in the 2011/12 season.

**Table 2:** The number of Influenza A positive cases by age group in the exponential period of each season.

	2011/12 season	2012/13 season
	No. (%)	No. (%)
Age Categories		
Preschool	272 (26.0)	139 (15.0)
Primary School	329 (31.4)	146 (15.8)
Junior High School	69 (6.6)	104 (11.2)
High School	47 (4.5)	60 (6.5)
Adults	329 (31.5)	478 (51.6)
Total	1046(100)	927(100)

The patterns among different age groups were compatible with those for the whole season (Table 1) except for junior high school students. The proportions of cases in preschool and primary school to the total number of cases in the exponential period were 26.0% and 31.4% in the 2011/12 season, and 15.0% and 15.8% in the 2012/13 season, respectively. These percentages were higher than the percentages of total cumulative numbers of both age groups in the two seasons as shown in Table 1.

**Fig 3:**
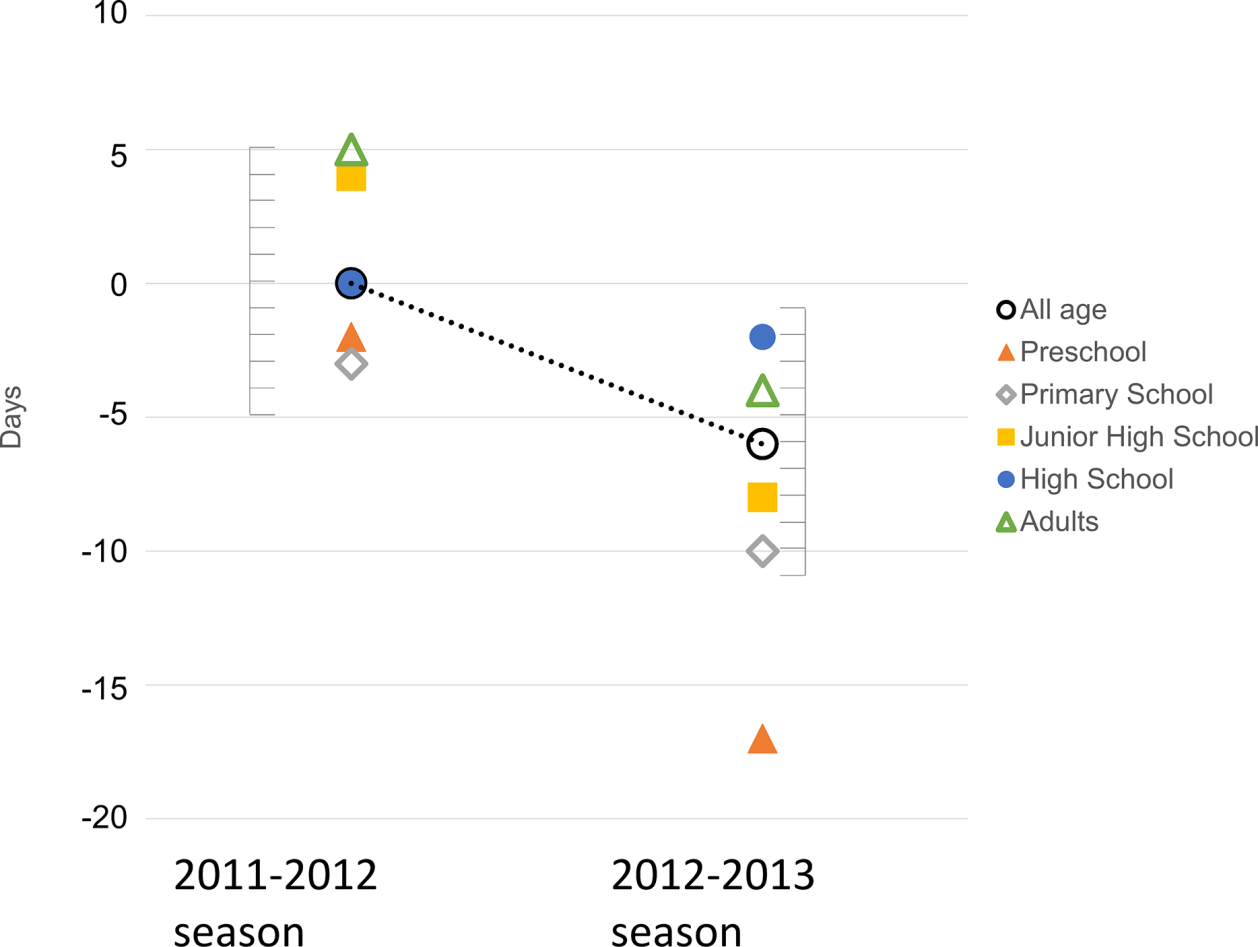
The distributions of Epidemic Midpoint (EM) by age groups in 2011/2012 and 2012/2013 seasons. *Day 0 was set as February 8.

The epidemic midpoint (EM) for the 2011/12 season was February 8, 2012 and that for the 2012/13 season was February 2, 2013 (Fig 3). The EM of all ages shifted by -6 days in 2012/13 without statistical significance (p = 0.49). When the epidemic midpoint of each age group was compared with the overall EM, the differences for preschool, primary school, junior high school, high school, and adults were -2, -3, 4, 0 and 5 days, respectively, in the 2011/2012 season, and -11, -4, -2, 4 and 2 days, respectively, in the 2012/13 season. Epidemic midpoints for both preschool and primary school groups were observed prior to the overall EM in the 2011/12 and the 2012/13 seasons, while EM for adult group was observed after the overall EM for both seasons. Except for preschool in the 2012/13 season, those differences were observed within ±5 days.

Multiple linear regression analysis was conducted to investigate the association between GRs and incidences in preschool, primary schools and others by primary school district as shown in Table 3.

**Table 3:** Result of multiple linear regression analysis between growth rate (GR) and the incidence of influenza cases in age group (preschool, primary school and others) by school districts (all 18 primary school districts in Odate City were included in the model).

Season;model	Adjusted R2	Variables	Estimated Coefficient (95% C.I.*)
2011/12;model 1	0.20	Incidence of preschool cases	0.043 (-0.447, 0.532)
	0.20	Incidence of primary school cases	0.378 (0.015, 0.741)
	0.20	Incidence of cases in other groups	0.047 (-0.44, 0.534)
2012/13;model 1	0.24	Incidence of preschool cases	-0.102 (-0.606, 0.402)
	0.24	Incidence of primary school cases	0.331 (0.081, 0.581)
	0.24	Incidence of cases in other groups	-0.01 (-0.311, 0.290)
2012/13;model 2	0.37	Incidence of preschool cases in 2012/13 season	-0.037 (-0.538, 0.464)
	0.37	Incidence of primary school cases in 2012/13 season	0.454 (0.145, 0.762)
	0.37	Incidence of cases in other groups in 2012/13 season	-0.002 (-0.295, 0.291)
	0.37	Number of primary school cases in 2011/12 season	0.0008 (-0.00006, 0.002)

* C.I.: Confidence interval.

The estimated coefficient for the incidence of primary school was 0.378 (95% C.I. 0.015 to 0.741) in 2011/2012 season and 0.331 (95% C.I. 0.081 to 0.581) in 2012/13 season but the coefficients for incidence of preschool and others were not significant in the model. Further, we included the number of primary school cases in the 2011/12 season into the linear regression model of 2012/13 season but that variable did not contribute to the improvement of the fitness (Adjusted R^2^ = 0.37). And all of VIFs were less than 10. Therefore, significant multicollinearity was not found in those variables.

We collected the influenza vaccination status only for 2012/13 season cases. In that season, overall vaccination rate against influenza was 28.3%. The rates were 41.4% for preschool children, 47.3% for primary school children and 36.3% for junior high school children. Those among high school students and adults were 23.3% and 21.4%, respectively.

## Discussion

Enhanced influenza surveillance was implemented in Odate City, Japan in the 2011/12 and the 2012/13 seasons. In these two consecutive seasons, influenza A(H3N2) with similar antigenic properties was the predominant influenza A subtype. We compared the epidemiological characteristics especially transmission dynamics in the two seasons. Consecutive A(H3N2)-predominant influenza seasons might cause a significant difference in epidemiological characteristics between the two seasons.

A significant decline in the total number of cases was observed in the 2012/13 season. This decreasing trend was also reported at the national level in Japan [13,14]. The analysis by age groups revealed a significant decline in the number of cases in preschool and primary school children while the number of cases in the high school and adult age group increased. In these two seasons, influenza A(H3N2) was the predominant influenza A subtype and there were no major antigenic changes reported among the circulating influenza A(H3N2) strains between the two seasons [13, 14]. The subtype-specific immunity against influenza A(H3N2) in which immunity from infections in previous seasons can reduce the risk of H3N2 re-infection in subsequent seasons [6,21–25]. A study in the Netherlands found a significant contribution of depletion of susceptible population to the trend of influenza-like illness epidemics across seasons [4]. In our study population, the outbreak of influenza A(H3N2) in the 2011/12 season might have led to the depletion of susceptible population in the 2012/13 season, which resulted in different epidemiological patterns in 2012/13. The decrease in the total number of influenza A cases in the 2012/13 season was mainly due to the decline in the number of cases in preschool and primary school children. In these age groups in the 2011/12 season, the attack rates (AR) were much higher than the other age groups (Table 1). This also supports a possibility that depletion of susceptible population, especially in preschool and primary school children might be responsible for the decreased trend of overall influenza A infection in the 2012/13 season. The depletion of susceptible population might cause the decrease in the influenza A cases of preschool and primary school children in the exponential period (Table 2), then led to the significant reduction of GR in the 2012/13 season.

The epidemic midpoint is a simple estimation of the timing of maximum activity of an epidemic [15,26]. The observed earlier EM of preschool and primary school-age groups (Fig 3) indicate earlier peaks of influenza A activity in these age groups compared with the others. Earlier EM for preschool children in the 2012/13 season might be attributable to the final size of influenza cases among preschool children in 2012/13 season, which was smaller than that in 2011/12 season (Table 1). But the cause of this discrepancy was unclear. With an assumption of an equal growth rate in different age groups, EM is theoretically affected by the timing of case introduction as well as the final size of population. Fig 1B showed the earlier introduction of influenza A in preschool and primary school children while the number of cases decreased rapidly during the period of -10 to 0 days from the epidemic midpoint. This implied that the spread of influenza among those populations did not connect to other populations.

Studies about epidemic midpoints of seasonal influenza epidemics documented the earlier EM among school-age children but the earliest was found in slightly different groups such as children aged between 10−19 years in Canada [15] and 12−18 years in the U.S [26]. The difference between this study and previous studies may be due to study methods and final size of influenza cases, which can explain the differences of age groups with the earliest EM. Specifically, only 2 consecutive seasons with H3N2 were included in this study while previous studies in U.S. and Canada analyzed the data for more than 5 seasons. The final size of cases was approximately 2,000 per season in this study while more than 5,000 cases in the Canadian study.

Our regression analysis indicated that incidence of primary school children was a significant determinant for the overall GR in the 2011/12 and 2012/13 seasons. This suggests that school children have an important role in the influenza transmission during the exponential period. We thought that the decrease in the influenza A cases in the 2012/13 season might be due to the depletion of susceptible population by infection in the 2011/12 season especially in primary school children. But the total number of primary school cases in the 2011/12 season did not improve the fitting of linear model with the GR in the 2012/13 season. The unimproved fitting might be explained by the heterogeneity of influenza A introduction and transmission among different school districts, rather than excluding a possibility that depletion of susceptible population was associated with decreased transmission in 2012/13 season. But it is not clear why the incidence of preschool was not significantly related with GR by school districts. A further investigation on the mixing pattern between preschool and primary school children may provide a clue for such a pattern.

All of these findings suggested that children play a major role in both introducing and/or spreading influenza infection into the community during the exponential period. It should be noted that Odate City has highly ageing population. The proportion of those aged > 65 years is 30.4%, which is higher than the national average (21.9%) and the proportion of those < 20 years is 15.0%, which is lower than the national average (17.9%). Even in such ageing community, school-age children are playing an important role in influenza transmission. In 2012/13 season, majority of influenza A cases occurred in adults (58%) (Table 1). But our data indicated that school-age children were also a driving force for community transmission of influenza A during early phase of epidemic in this season. Although our study indicated that children in preschool and primary school might have an important role for community transmission of influenza, there are several limitations to our datasets. First, we assumed all influenza A positive cases were due to influenza A(H3N2) without laboratory confirmation. Influenza diagnosis was based on the rapid diagnostic assays, which showed 66.7 to 95% of sensitivity and 88 to 95% of specificity [27, 28]. There were 2 influenza A (H1N1)pdm09 out of 40 influenza A positive samples reported in the 2012/13 season in Akita Prefecture where Odate city is located. It is possible that some of influenza A positive cases were infected with influenza A (H1N1)pdm09, however the number was believed to be minimal. Second, we could not include the entire data of seasonal vaccination among residents. Seasonal vaccination status and the immunogenicity of seasonal dominant influenza strain can affect the epidemiological characteristics of influenza in the community. Complete data on vaccination status in study population may provide a more precise model to investigate the effect of vaccination, but the vaccine coverage data for whole population was not available in this study. Third, we analyzed the data from enhanced surveillance which potentially misses some cases. Although we included 22 out of 36 outpatient clinics in Odate City, some patients might have visited clinics which did not participate in the study or some might not have visited any health facilities. The consultation rates might be particularly low in adults and elderlies [12] and this may affect the analysis of this study. We also did not consider asymptomatic infections which may account for as high as 33% of all influenza A infections [29].

Despite those limitations, our surveillance consisted of majority of clinics. It was also shown that more than 95% of patients with influenza-like symptoms sought consultation within 2 days [12] when the viral load is supposed to be high enough to be detected by rapid kits. Thus, this dataset provided a good representative of influenza A cases among the study population.

In conclusion, our findings strengthen the evidence that school-age children play an important role as a driving force for community transmission of influenza A(H3N2).This information is useful for influenza control and prevention in the community.

## Supporting Information

S1 Table Data of influenza A positive cases. (XLS)

## References

[1] World Health Organization (2014) Influenza (Seasonal) Fact sheet No. 211. Available: http://www.who.int/mediacentre/factsheets/fs211/en/. Accessed 15 September 2014.

[2] Alexander ME, Kobes R (2011) Effects of vaccination and population structure on influenza epidemic spread in the presence of two circulating strains. BMC Public Health 11 Suppl 1: S8 10.1186/1471-2458-11-S1-S8 21356137 PMC3317581

[3] Fantoni A, Arena C, Corrias L, Salez N, de Lamballerie XN, Amoros JP, et al (2014) Genetic drift of influenza A(H3N2) viruses during two consecutive seasons in 2011–2013 in Corsica, France. J Med Virol 86: 585–591. 10.1002/jmv.23745 24105757

[4] te Beest DE, van Boven M, Hooiveld M, van den Dool C, Wallinga J (2013) Driving factors of influenza transmission in the Netherlands. Am J Epidemiol 178: 1469–1477. 10.1093/aje/kwt132 24029683

[5] Lunelli A, Rizzo C, Puzelli S, Bella A, Montomoli E, Rota MC, et al (2013) Understanding the dynamics of seasonal influenza in Italy: incidence, transmissibility and population susceptibility in a 9-year period. Influenza Other Respir Viruses 7: 286–295. 10.1111/j.1750-2659.2012.00388.x 22694182 PMC5779816

[6] Cowling BJ, Perera RA, Fang VJ, Chan KH, Wai W, So HC, et al. (2014) Incidence of influenza virus infections in children in Hong Kong in a three year randomised placebo-controlled vaccine study, 2009–12. Clin Infect Dis. 10.1093/cid/ciu356 24825868

[7] Brownstein JS, Kleinman KP, Mandl KD (2005) Identifying pediatric age groups for influenza vaccination using a real-time regional surveillance system. Am J Epidemiol 162: 686–693. 16107568 10.1093/aje/kwi257 PMC1266301

[8] Monto AS, Davenport FM, Napier JA, Francis T (1969) Effect of vaccination of a school-age population upon the course of an A2-Hong Kong influenza epidemic. Bull World Health Organ 41: 537–542. 5309469 PMC2427727

[9] Miller E, Hoschler K, Hardelid P, Stanford E, Andrews N, Zambon M (2010) Incidence of 2009 pandemic influenza A H1N1 infection in England: a cross-sectional serological study. Lancet 375: 1100–1108. 10.1016/S0140-6736(09)62126-7 20096450

[10] Kucharski AJ, Kwok KO, Wei VW, Cowling BJ, Read JM, Lessler J, et al (2014) The contribution of social behaviour to the transmission of influenza A in a human population. PLoS Pathog 10: e1004206 10.1371/journal.ppat.1004206 24968312 PMC4072802

[11] National Instutute of Infectious Disease (2014) Situation Update of Influenza Surveillance. Available: http://www.nih.go.jp/niid/images/idsc/disease/influ/fludoco1314.pdf. Accessed 15 September 2014.

[12] Huo X, Kamigaki T, Mimura S, Takahashi Y, Oshitani H (2014) Analysis of medical consultation interval between the symptom onset and consultation observed in multiple medical facilities in Odate city, Japan, 2011/2012 and 2012/2013 seasons. J Infect Chemother. 10.1016/j.jiac.2014.02.005 24725622

[13] National Institute of Infectious Diseases, Japan (2012) 2011/12 influenza season, Japan. Infectious Agents Surveillance Report 33: 285–287. Available: http://www.nih.go.jp/niid/en/iasr-vol33-e/2930-inx393-e.html. Accessed 15 September 2014.

[14] National Institute of Infectious Diseases, Japan (2013) 2012/13 influenza season, Japan. Infectious Agents Surveillance Report 34: 325–327. Available: http://www.nih.go.jp/niid/en/iasr-vol34-e/865-iasr/4124-tpc405.html. Accessed 15 September 2014.

[15] Schanzer D, Vachon J, Pelletier L (2011) Age-specific differences in influenza A epidemic curves: do children drive the spread of influenza epidemics? Am J Epidemiol 174: 109–117. 10.1093/aje/kwr037 21602300 PMC3119537

[16] Nishiura H, Chowell G, Safan M, Castillo-Chavez C (2010) Pros and cons of estimating the reproduction number from early epidemic growth rate of influenza A (H1N1) 2009. Theor Biol Med Model 7: 1 10.1186/1742-4682-7-1 20056004 PMC2821365

[17] Wallinga J, Lipsitch M (2007) How generation intervals shape the relationship between growth rates and reproductive numbers. Proc Biol Sci 274: 599–604. 17476782 10.1098/rspb.2006.3754 PMC1766383

[18] Biggerstaff M, Cauchemez S, Reed C, Gambhir M, Finelli L (2014) Estimates of the reproduction number for seasonal, pandemic, and zoonotic influenza: a systematic review of the literature. BMC Infect Dis 14: 480 10.1186/1471-2334-14-480 25186370 PMC4169819

[19] Opatowski L, Fraser C, Griffin J, de Silva E, Van Kerkhove MD, Lyons EJ, et al (2011) Transmission characteristics of the 2009 H1N1 influenza pandemic: comparison of 8 Southern hemisphere countries. PLoS Pathog 7: e1002225 10.1371/journal.ppat.1002225 21909272 PMC3164643

[20] Ministry of Land, Inflastructure, Transport and Tourism (2010) Map of primary school districts in Japan. Available: http://www.mlit.go.jp/kokudoseisaku/kokudojoho.html. Accessed 15 September 2014.

[21] Hoskins TW, Davies JR, Smith AJ, Allchin A, Miller CL, Pollock TM (1976) Influenza at Christ's Hospital: 3, 1974. Lancet 1: 105–108. 54631 10.1016/s0140-6736(76)93151-2

[22] Gill PW, Murphy AM (1977) Naturally acquired immunity to influenza type A: a further prospective study. Med J Aust 2: 761–765. 611373 10.5694/j.1326-5377.1977.tb99276.x

[23] Gill PW, Murphy AM (1985) Naturally acquired immunity to influenza type A. Lessons from two coexisting subtypes. Med J Aust 142: 94–98. 3965918 10.5694/j.1326-5377.1985.tb133042.x

[24] Grilli EA, Davies JR, Smith AJ (1986) Infection with influenza A H1N1. 1. Production and persistence of antibody. J Hyg (Lond) 96: 335–343. 3701045 10.1017/s0022172400066080 PMC2129637

[25] Ambrose CS, Wu X (2011) Evidence of homosubtypic but not heterosubtypic immunity in young children following wild-type influenza illness. Pediatr Infect Dis J 30: 900–901. 10.1097/INF.0b013e31821ffe43 21577174

[26] Stockmann C, Pavia AT, Hersh AL, Spigarelli MG, Castle B, Korgenski K, et al (2014) Age-Specific Patterns of Influenza Activity in Utah: Do Older School Age Children Drive the Epidemic? J Pediatric Infect Dis Soc 3: 163–167. 24872880 10.1093/jpids/pit004 PMC4036424

[27] Ruest A, Michaud S, Deslandes S, Frost EH (2003) Comparison of the Directigen flu A+B test, the QuickVue influenza test, and clinical case definition to viral culture and reverse transcription-PCR for rapid diagnosis of influenza virus infection. J Clin Microbiol 41: 3487–3493. 12904343 10.1128/JCM.41.8.3487-3493.2003 PMC179849

[28] Chan KH, Chan KM, Ho YL, Lam YP, Tong HL, Poon LL, et al (2012) Quantitative analysis of four rapid antigen assays for detection of pandemic H1N1 2009 compared with seasonal H1N1 and H3N2 influenza A viruses on nasopharyngeal aspirates from patients with influenza. J Virol Methods 186: 184–188. 10.1016/j.jviromet.2012.09.001 22989408

[29] Li T, Liu Y, Di B, Wang M, Shen J, Zhang Y, et al (2011) Epidemiological investigation of an outbreak of pandemic influenza A (H1N1) 2009 in a boarding school: serological analysis of 1570 cases. J Clin Virol 50: 235–239. 10.1016/j.jcv.2010.11.012 21195022

